# Health Supports Learning: Academic Outcomes Improved by the School‐Based Asthma Therapy Program

**DOI:** 10.1111/josh.70253

**Published:** 2026-10-06

**Authors:** Kimberly Arcoleo, Holly McGregor, Phoebe Marx, Elizabeth Allen, Mary Kay Irwin, Ian Dela Cruz, Jill S. Halterman

**Affiliations:** ^1^ College of Nursing Michigan State University East Lansing Michigan USA; ^2^ School of Medicine & Dentistry University of Rochester New York New York USA; ^3^ School Health Services Nationwide Children's Hospital Columbus Ohio USA

**Keywords:** absenteeism, anti‐asthmatic drugs, asthma, problem behavior

## Abstract

**Background:**

School nurses are vital to childhood asthma management. Although school‐based asthma therapy (SBAT) improves clinical outcomes, its academic impact remains unclear. SBAT reduces barriers to guideline‐based care through directly observed preventive medication therapy for children with persistent, poorly controlled asthma.

**Methods:**

Retrospective academic record review (2013–2019) for 552 SBAT‐enrolled children in a large urban district. Outcomes included missed instructional time, behavioral incidents, and grades compared across 4 quarters pre‐ and post‐enrollment, stratified by school level, sex, race/ethnicity, and SES.

**Results:**

Unexcused hours decreased significantly post‐SBAT: 17% elementary; 53% middle/high school, with no sex or race/ethnicity differences. Excused hours decreased 27% among middle/high school students. Level 2 incidents decreased 32% in elementary and 34% in middle/high school students. Females had more excused hours and Level 1/2 behavioral incidents than males, while Black elementary students had fewer Level 1/2 incidents than Whites. Grades did not change significantly.

**Implications for School Health, Policy, Practice, and Equity:**

SBAT has potential as an equity‐promoting school healthcare practice to mitigate disparities in asthma morbidity, absenteeism, and school engagement among students in disadvantaged communities.

**Conclusions:**

SBAT highlights potential educational benefits, supporting integration of guideline‐based asthma management into routine school health services.

## 1 Introduction

Asthma affects more than 4 million US children leading to significant morbidity and cost [1]. Uncontrolled asthma (> 40% of children) [2] causes morbidity from recurrent symptoms, activity limitations, school absenteeism, and caregiver work loss. For children with persistent asthma [3], adherence to effective, guideline‐based anti‐inflammatory medication is poor [4, 5, 6]. Children living in impoverished rural or urban areas are disproportionately affected by asthma and less likely to receive appropriate care, leading to preventable morbidity [4, 5, 6].

School‐based directly observed therapy (DOT) programs represent a promising strategy for asthma management because of the ability to optimize children's care in the setting in which they spend most of their day [7, 8], and reach children at increased risk for poor outcomes even if they have limited contact with the health care system [9, 10, 11]. While all US states allow children to carry their asthma medications at school under the ASTHMA Act [12], this has almost exclusively consisted of quick relief inhalers. Use of controller medications during the school day is uncommon and inconsistently implemented. Our school‐based asthma therapy (SBAT) program focuses on children with persistent, poorly controlled asthma, reduces multiple barriers to guideline‐based asthma care, and improves outcomes through school‐based DOT of effective preventive anti‐inflammatory medications [9, 10, 11, 13]. By delivering daily preventive medications in school, adherence can be assured on days the child attends school, and because schools (even without full‐time nurses) already routinely provide medications for other conditions such as attention deficit disorder, the administration of daily preventive asthma medications represents a conceptually simple practice change to improve adherence. Embedding guideline‐based asthma therapy into routine school workflows addresses a critical evidence‐to‐practice gap with direct relevance to children, caregivers, schools, and health systems. Although there is a growing body of evidence demonstrating improved clinical outcomes for children participating in school‐based asthma therapy, little is known about the impact on academic outcomes. Salazar, Tarwala and Reznik [14] conducted a literature review of school‐based DOT programs and reported results for nine studies that met the inclusion criteria. They concluded that supervised school‐based asthma medication programs have demonstrated broad benefits, including improved medication adherence and symptom control, fewer exacerbations and healthcare visits, reduced school absenteeism and activity limitations, better quality of life, and improved pulmonary outcomes. Seven of the nine studies were randomized controlled trials, one was a pre−/post‐test design, and one a retrospective study and although school absenteeism was reported in four studies (three of those studies by a member of our team) [11, 15, 16], none reported absenteeism by excused versus unexcused, academic grades, or behavioral incidents. To the best of our knowledge, no other study has been able to assess academic grades or behavioral incidents in addition to missed instructional time by excused and unexcused absences.

We are reporting academic outcome findings from a large‐scale real‐world implementation of SBAT in Columbus, OH, the largest school district in Ohio. Approximately 65% of students in the Columbus City School District miss at least 10% of instructional time—whether excused or unexcused—and chronic absenteeism in the district is twice the state average and ˜ four times the national average [17]. The goal of these analyses was to investigate the impact of a real‐world implementation of the SBAT program on children's missed instructional time, behavioral incidents, and grades for the 1‐year prior to SBAT‐enrollment and the 1‐year post‐enrollment. Our hypotheses were that the SBAT program would reduce missed instructional time and behavioral incidents and improve academic grades.

## 2 Methods

### 2.1 Design and Participants

The main implementation evaluation study was reviewed and approved by the Nationwide Children's Hospital Institutional Review Board (IRB #00000236; approved April 22, 2019). Reliance agreements were established with Michigan State University, University of Rochester, and University at Buffalo. All caregivers provided written informed consent for their and their children's participation in SBAT and a separate consent for the implementation evaluation study and children provided assent/consent based on their age. For these retrospective record analyses, no data were collected from caregivers or children. A Data Sharing Agreement between Columbus City School District and Nationwide Children's Hospital was executed allowing access to students' academic records. Academic records for 552 children who were enrolled in the SBAT program and attended Columbus City School District for four quarters pre‐SBAT enrollment and four quarters post‐enrollment were abstracted. Data from 2013 to 2019 were abstracted from the Infinite Campus student information system. Due to the Covid‐19 pandemic, schools closed in March 2020 and re‐opened in a hybrid format August 2020. A 2023 systematic review of the impact of COVID‐19 lockdown on children's asthma control revealed that contrary to the original fear of worse outcomes for these children that the lockdown was associated with better asthma control, primarily due to reduced trigger exposure and improved management [18]. In addition, students did not return to full‐time, in‐person instruction until the 2021–2022 academic year. The first year of post‐Covid in‐person instruction presented many challenges for schools and students. Chronic absenteeism (defined as missing 10% or more of school days) increased over 13 percentage points between 2018–2019 and 2021–2022—a 91% increase [19]. These factors combined with the fact that we were in the process of rebuilding the SBAT program, a decision was made to not abstract data from the 2021–2022 academic year. In addition, grant funding ended in 2023 and thus, we were unable to abstract post‐enrollment data for the 2023–2024 academic year. The variables extracted are detailed below. Each child contributed 1 year of pre‐SBAT and 1 year of post‐SBAT data.

### 2.2 Instrumentation

#### 2.2.1 Child Demographics

From the Infinite Campus database, the child's sex, race/ethnicity, and language spoken were extracted. Race/ethnicity was coded as White, Black, Hispanic, Asian, or Multi‐racial. Language was coded as English, Spanish, Somali, or Other. Country of birth and citizenship status were not abstracted due to privacy considerations. From children's medical records, we abstracted the Child Opportunity Index (COI), a proxy for SES. The COI is a multidimensional measure of neighborhood conditions that support child health and development, incorporating 29 indicators across educational, health and environmental, and social and economic domains. Domain‐specific measures were obtained at the finest available geographic resolution and aggregated to the census tract level using annual five‐year moving averages. Each participant was assigned a COI score based on the census tract of residence. Overall COI scores are derived from standardized composites of the domain‐specific indicators and range from 1 to 100, with higher scores indicating greater opportunity. Scores are categorized as “very low”, “low”, “moderate”, “high”, and “very high.” [20].

### 2.3 Outcome Measures

#### 2.3.1 Missed Instructional Hours

Required instructional time differs between elementary and middle/high school students, thus we calculated missed instructional hours separately for each group [21]. Absences and missed instructional time were selected for the four academic quarters pre‐SBAT enrollment and four quarters post‐enrollment. Record year, quarter, absence date, absent minutes, scheduled minutes, and whether the absence was excused or unexcused were extracted. Per the school districts' attendance codebook, excused absences are defined as a “Verified student absence. Student excuse note required to document absence.” Unexcused absences are defined as “Student absences that have not been verified. School office has not received parent excuse note, documentation or notification as to the reason for absence or the reason for absence is not excusable as per Board Policy.” For each student, total missed instructional minutes were calculated and two averages created: (1) Average number of absence minutes for the four quarters prior to enrollment in SBAT and (2) Average number of absence minutes for the four quarters after SBAT enrollment. Minutes were then converted to hours by dividing by 60. This procedure was done separately for excused and unexcused time. Students with no absences in either period were coded as “0.”

#### 2.3.2 Behavioral Incidents

To investigate the impact on behavioral incidents the following variables were abstracted: behavioral incident date, incident name, severity level, and incident resolution. Similar to absence data, behavioral incident data were abstracted for the four academic quarters pre‐SBAT enrollment and the four academic quarters post‐enrollment. There are three severity levels of behavioral incidents: Level I = minor offenses such as disruptive behavior or profanity; Level II = repeated Level I offenses or serious misconduct (e.g., bullying, fighting); and Level III = repeated Level I or Level II offenses, illegal and/or serious misconduct, or life or health threatening offenses (such as dangerous weapon, sexual offense). Averages for Level 1 and Level 2 incidents were computed overall and separately for elementary and middle/high school students. Students with no behavioral incidents pre‐ and post‐SBAT enrollment were included and coded as “0.”

#### 2.3.3 Grades

Because there is no alpha‐numeric equivalent for the grades used in elementary school, the analyses were restricted to middle and high school grades for four core courses: English Language Arts (ELA), Math, Science, and Social Studies. Grades were abstracted for the four academic quarters pre‐ and four academic quarters post‐SBAT enrollment. Grades were reported as letter grades and then converted to numeric values following the Ohio Department of Education and Workforce's “GPA Calculation Chart.” [22] Once numeric values for each grade were generated, these values were averaged within each academic quarter and course to create quarter‐level grade averages. It should be noted that the calculations used do not produce grade point average (GPA), as GPA is the average of grades in a quarter weighted by the number of credits a student took in that quarter. For these analyses, we analyzed the unweighted grade averages by quarter.

### 2.4 Procedure

Caregivers and children completed informed consent/assent for participation in the SBAT program. These consent documents also allowed the SBAT team to have access to children's medical and academic records for program evaluation. Once a child enrolls in SBAT, this information is communicated to the school district's Student Records department. A flag in the Infinite Campus system is entered into the student's record, which follows the student even if there is a change in schools while enrolled in the program. Data (child demographics, record year, school, grade, absences, behavioral incidents, and academic grades) (middle/high school only) for all flagged children 2013–2019 were downloaded into Excel files. These files were then verified against the original SBAT enrollment files to ensure that all flagged children were actually enrolled in SBAT. After verification, the Excel files were then converted to SAS datasets for analyses.

### 2.5 Statistical Analysis

Total, excused, and unexcused missed instructional hours, behavioral incidents, and grades were analyzed for the whole sample, elementary and middle/high school students. A repeated measures approach (SAS PROC MIXED), which controlled for age and examined potential differences by race/ethnicity, sex, and SES, was used. Due to the high proportion of zero Level I and Level II behavioral incidents, a logistic regression was run specifying a logit link and the Firth correction. The Firth correction reduces the bias in the maximum likelihood estimates due to the preponderance of zeros. As a result of several small cell sizes, race/ethnicity was collapsed to White, Black, and Latino/Multi‐Racial. Categorial independent variables were included as fixed effects in all models and the following categories (coded as “0”) specified as the reference category for each: sex = male; race/ethnicity = White; and Child Opportunity Index = Very High.

## 3 Results

There were 437 elementary and 115 middle/high school student records abstracted. For the elementary students, there were 1660 total absences and 447 behavioral incidents pulled. For the middle/high school students, 716 absences, 168 behavioral incidents, and 148 grades were obtained. Table 1 presents the sample characteristics for the elementary and middle/high school samples and examination of baseline differences. Middle/high school students had higher baseline absences (both types) and Level 2 behavioral incidents. Figure 1 and Table 2 show the results for total, unexcused, and excused missed instructional time by group. Statistically significant reductions in average number of total and unexcused hours from pre‐ to post‐SBAT enrollment for the whole sample, elementary students, and middle/high school students were noted. Overall, there was a 10% and 84% reduction in total missed instructional hours for elementary and middle/high school students, respectively. Unexcused missed instructional hours decreased significantly by 17% while excused missed instructional hours increased by 12% (not statistically significant). Middle/high school students had 27% and 53% reductions in excused and unexcused hours, respectively. There were no differences in total missed instructional time by sex or ethnicity. Middle/high school students in the Low COI group had 43% fewer missed total and 40% unexcused instructional hours compared to the Very Low group. Elementary children in the Moderate COI group had 38% fewer excused hours than the Very Low group. Middle/high school females had 38% more excused hours than males.

**TABLE 1:** Pre‐SBAT enrollment sample characteristics.

Characteristic	Elementary (*n* = 437)	Middle/high school (*n* = 115)	*χ* ^2^ *p* value^a^
	*N* (%)	*N* (%)	
Sex			0.19
Male	265 (60.64)	62 (53.91)	—
Female	172 (39.36)	53 (46.09)	
Race/ethnicity			0.72
White	51 (11.67)	16 (13.91)	—
Black	336 (76.89)	88 (76.52)	
Latino/bi‐racial	50 (11.44)	11 (9.57)	
Child opportunity index (proxy for SES)			0.19
Very low	308 (71.96)	94 (81.74)	
Low	58 (13.55)	13 (11.30)	
Moderate	21 (4.91)	4 (3.48)	
High	15 (3.50)	1 (0.87)	
Very high	26 (6.07)	3 (2.61)	

	Mean (SD)	Mean (SD)	*t*‐test *p* value^a^
Age	7.79 (2.18)	11.87 (3.05)	< 0.0001
Average absence hours			
Unexcused	**23.03 (21.17)**	**37.56 (34.24)**	**< 0.001**
Excused	**8.26 (8.71)**	**11.14 (13.75)**	**0.006**
Behavioral incidents			
Level 1	0.22 (0.41)	0.25 (0.43)	0.49
Level 2	**0.29 (0.45)**	**0.44 (0.50)**	**0.002**

^a^ Bolded values represent statistically significant differences between elementary and middle/high school students.

**FIGURE 1:**
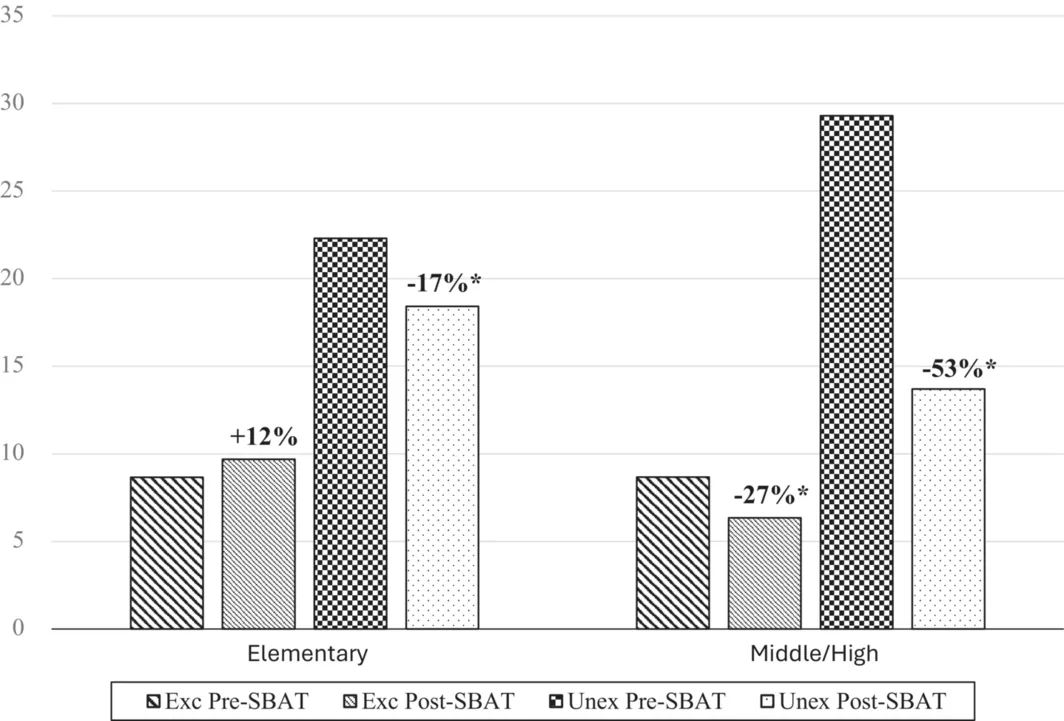
Average # Missed instructional hours/year.

**TABLE 2:** Model results for total, unexcused, and excused missed instructional time: 1 year pre‐ and 1 year post‐SBAT enrollment.

Variable	Average total missed instructional hours—elementary school			Average total missed instructional hours—middle/high school		
	LSMeans (SE)	Δ (95% CI)^a^	*p*‐Value^a^	LSMeans (SE)	Δ (95% CI)^a^	*p*‐Value^a^
Time period						
Pre‐SBAT Enrollment	15.59 (1.03)	—	—	18.38 (4.39)	—	—
Post‐SBAT Enrollment	14.06 (1.03)	**1.52 (−0.02, 3.07)**	**0.05**	8.95 (4.39)	**9.43 (5.22, 13.64)**	**< 0.0001**
Race/ethnicity						
White (Ref)	13.36 (1.49)	—	—	9.37 (5.03)	—	—
Black	14.89 (0.90)	1.53 (1.51)	0.31	10.71 (4.03)	1.34 (−5.45, 8.13)	0.70
Latino/multi‐racial	16.22 (1.59)	2.86 (1.99)	0.15	12.63 (4.89)	3.26 (−6.68, 13.20)	0.52
Sex						
Male (Ref)	14.99 (1.01)	—	—	10.21 (4.05)	—	—
Female	14.66 (1.12)	0.33 (0.99)	0.74	11.59 (4.21)	−1.39 (−5.90, 3.13)	0.54
Child opportunity index						
Very low (Ref)	16.32 (1.97)	—	—	18.23 (1.91)	—	—
Low	12.51 (2.59)	3.81 (−2.48, 10.11)	0.23	10.48 (3.23)	**−7.76 (−14.47, −1.04)**	**0.02**
Moderate	17.92 (2.29)	−1.60 (−7.36, 4.16)	0.59	8.44 (9.24)	−9.79 (−27.82, 8.24)	0.29
High	11.95 (1.33)	4.37 (−0.19, 8.94)	0.06	1.67 (15.14)	−16.56 (−46.97, 13.84)	0.28
Very high	15.43 (0.79)	0.89 (−3.06, 4.85)	0.66	15.68 (7.35)	−2.55 (−16.90, 11.79)	0.73
Age	*F* = 0.43		0.51	*F* = 0.49		0.49

Variable	Average missed instructional hours unexcused‐elementary school			Average missed instructional hours unexcused‐middle/high school		
	LSMeans (SE)	Δ (95% CI)^a^	*p*‐Value^a^	LSMeans (SE)	Δ (95% CI)^a^	*p*‐Value^a^
Time period						
Pre‐SBAT enrollment	**22.30 (1.66)**	—	—	29.30 (6.62)	—	—
Post‐SBAT Enrollment	**18.42 (1.66)**	**3.88 (1.74, 6.02)**	**0.0004**	13.70 (6.62)	**15.59 (11.15, 20.04)**	**< 0.0001**
Race/ethnicity						
White (Ref)	17.44 (2.46)	—	—	17.23 (8.46)	—	—
Black	21.38 (1.47)	3.94 (−0.93, 8.81)	0.11	22.98 (6.69)	5.75 (−5.97, 17.47)	0.33
Latino/multi‐racial	22.26 (2.64)	4.83 (−1.64, 11.29)	0.14	24.29 (8.02)	7.05 (−9.94, 24.05)	0.41
Sex						
Male (Ref)	20.88 (1.67)	—	—	21.74 (6.67)	—	—
Female	19.83 (1.86)	1.05 (−2.14, 4.23)	0.52	21.29 (6.93)	0.41 (−7.26, 8.08)	0.92
Child opportunity index						
Very low (Ref)	21.07 (3.25)	—	—	30.89 (3.20)	—	—
Low	16.43 (4.24)	4.64 (−5.70, 14.98)	0.38	18.52 (5.60)	**−12.37 (−24.12, −0.62)**	**0.04**
Moderate	28.77 (3.79)	−7.70 (−17.22, 1.82)	0.11	24.44 (13.28)	−6.46 (32.31, 19.40)	0.62
High	14.87 (2.22)	6.20 (−1.35, 13.74)	0.11	6.59 (26.12)	−24.31 (−76.76, 28.15)	0.36
Very high	20.65 (1.30)	0.41 (−6.10, 6.93)	0.90	27.07 (11.73)	−3.82 (−27.03, 19.38)	0.75
Age	*F* = 0.04	—	0.84	*F* = 22.52	—	< 0.0001

	Average missed instructional hours excused—elementary school (*n* = 131)			Average missed instructional hours excused—middle/high school		
Time period						
Pre‐SBAT enrollment	8.66 (0.78)	—	—	8.67 (2.90)	—	—
Post‐SBAT enrollment	9.69 (0.78)	−1.03 (−2.17, 0.11)	0.08	6.34 (2.90)	**2.33 (0.02, 4.63)**	**0.048**
Race/ethnicity						
White (Ref)	9.35 (1.14)	—	—	7.10 (3.55)	—	—
Black	8.09 (0.69)	−1.26 (−3.54, 1.01)	0.28	7.53 (2.87)	0.43 (−4.26, 5.11)	0.86
Latino/multi‐racial	10.08 (1.21)	0.73 (−2.26, 3.72)	0.63	7.88 (3.50)	0.78 (−6.13, 7.68)	0.82
Sex						
Male (Ref)	8.75 (0.77)			5.75 (2.90)		
Female	9.59 (0.86)	−0.84 (−2.33, 0.66)	0.27	9.26 (3.00)	**−3.51 (−6.65, −0.37)**	**0.03**
Child opportunity index						
Very low (Ref)	11.55 (1.50)	—	—	10.76 (1.30)	—	—
Low	8.33 (1.99)	3.22 (−1.60, 8.04)	0.19	6.74 (2.20)	−4.02 (−8.62, 0.58)	0.09
Moderate	7.14 (1.75)	**4.41 (0.01, 8.81)**	**0.049**	9.98 (7.38)	−0.78 (−15.25, 13.70)	0.92
High	9.11 (1.01)	2.44 (−1.04, 5.91)	0.17	2.27 (10.44)	−8.48 (−29.47, 12.51)	0.43
Very high	9.73 (0.61)	1.83 (−1.19, 4.84)	0.24	7.77 (5.32)	−2.99 (−13.33, 7.36)	0.57
Age	*F* = 2.39	—	0.12	*F* = 0.73	—	0.40

^a^ Represents the within‐group differences. Statistically significant differences are bolded.

As described above, behavioral incidents are categorized on a 3‐level scale, with Level 1 incidents being the less severe infractions and Level 3 incidents being more severe infractions. There were no instances of Level 3 infractions, so analyses focused only on Level 1 and 2 infractions.

Figure 2 and Table 3 reveal that across all students, there were no significant changes in Level 1 incidents. There were statistically significant decreases in the number of average Level 2 behavioral incidents pre‐to‐post SBAT enrollment for both groups (32% elementary; 34% middle/high school). Compared to White elementary students, Black students demonstrated significantly lower Level 1 (51%; *p* = 0.0007) and Level 2 (60%; *p* = 0.0008) behavioral incidents. No significant race/ethnicity differences were noted for middle/high school students for either level. Across both groups of students, females had significantly higher Level 1 (55% Elem, *p* < 0.0001; 22% MSHS, *p* = 0.05) and Level 2 (69% Elem, *p* < 0.0001; 48% MSHS, *p* = 0.002) incidents compared to their male counterparts. No significant differences in Level 1 incidents by COI group (SES proxy) were noted for elementary and middle/high school students. However, for elementary students only, compared to students in the Very Low Opportunity group, those in the High (55%; *p* = 0.04) and Very High (53%; *p* = 0.03) groups had significantly higher Level 2 behavioral incidents. Grade data (English Language Arts, Math, Science and Social Studies) were abstracted for 148 middle/high students for the four quarters prior to, and the four quarters after SBAT enrollment. There were no significant changes in any of the grades for the whole sample, sex, race/ethnicity, or SES.

**FIGURE 2:**
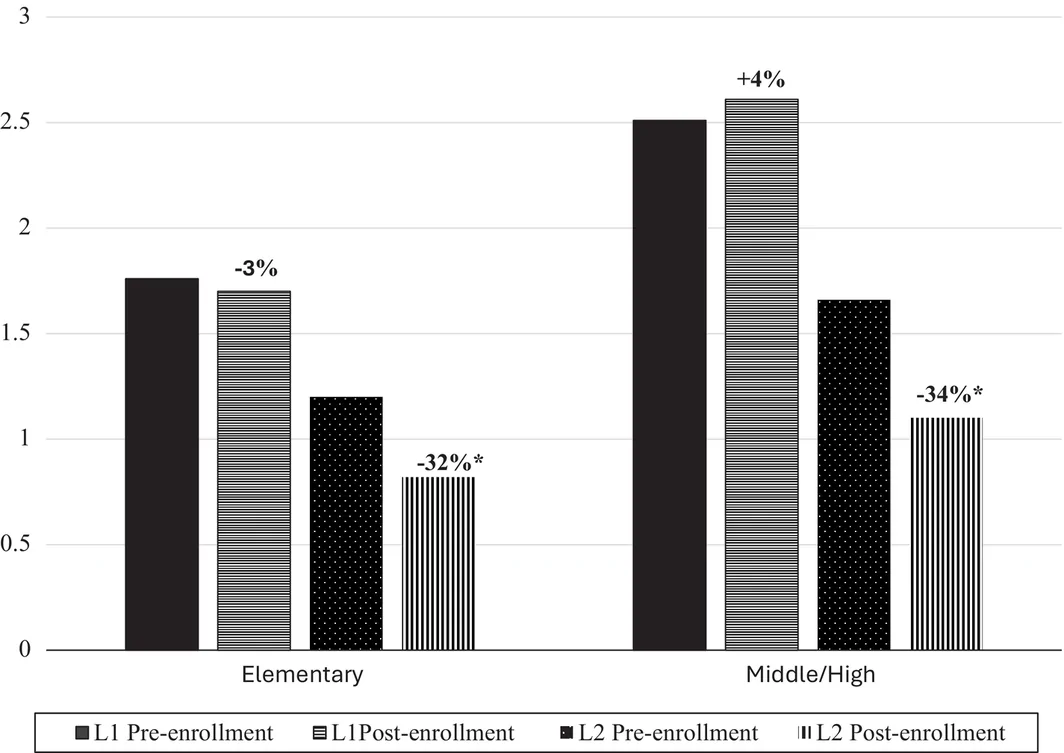
Average # Behavioral incidents/year.

**TABLE 3:** Model results for Level 1 and level behavioral incidents 1 year pre‐ and 1 year post‐SBAT enrollment.

Variable	Average Level 1 incidents—elementary school			Average Level 1 incidents—middle/high school		
	LSMeans (SE)	Δ (95% CI)^a^	*p*‐Value^a^	LSMeans (SE)	Δ (95% CI)^a^	*p*‐Value^a^
Time period						
Pre‐SBAT Enrollment	−1.76 (0.21)	—	—	−2.51 (0.67)	—	—
Post‐SBAT Enrollment	−1.70 (0.19)	−0.06 (0.67, 1.33)	0.75	−2.61 (0.68)	0.10 (0.60, 2.03)	0.75
Race/ethnicity						
White (Ref)	−2.42 (0.37)	—	—	−2.46 (0.74)	—	—
Black	−1.18 (0.16)	**1.24 (1.68, 7.05)**	**0.0007**	−1.96 (0.60)	0.50 (0.65, 4.18)	0.29
Latino/multi‐racial	−1.59 (0.31)	0.83 (0.95, 5.54)	0.06	−3.27 (1.02)	−0.81 (0.07, 2.96)	0.40
Sex						
Male (Ref)	−1.07 (0.19)	—	—	−2.25 (0.67)	—	—
Female	−2.39 (0.24)	**1.32 (2.52, 5.62)**	**< 0.0001**	−2.88 (0.69)	**0.63 (0.99, 3.56)**	**0.05**
Child opportunity index						
Very low (Ref)	−1.71 (0.37)	—	—	−3.38 (1.56)	—	—
Low	−1.47 (0.44)	−0.24 (0.27, 2.33)	0.66	—	—	—
Moderate	−1.49 (0.42)	−0.23 (0.25, 2.23)	0.66	−3.21 (1.71)	−0.16 (0.01, 72.36)	0.94
High	−1.93 (0.28)	0.22 (0.54, 2.90)	0.61	−2.18 (0.64)	−1.19 (0.01, 7.82)	0.47
Very high	−2.04 (0.18)	0.32 (0.68, 2.83)	0.37	−1.48 (0.33)	−1.90 (0.01, 3.17)	0.22
Age	***𝛘*** ^**2**^ **= 32.52**	—	**< 0.0001**	*𝛘* ^2^ = 0.37	—	0.55

	Average Level 2 incidents‐elementary school (*n* = 131)			Average Level 2 incidents‐middle/high school		
Time period						
Pre‐SBAT Enrollment	−1.20 (0.19)	—	—	−1.66 (0.54)	—	—
Post‐SBAT Enrollment	−0.82 (0.18)	**−0.38 (0.50, 0.93)**	**0.02**	−1.10 (0.52)	**−0.57 (0.33, 0.98)**	**0.04**
Race/ethnicity						
White (Ref)	−1.41 (0.28)	—	—	−1.43 (0.60)	—	—
Black	−0.56 (0.15)	**0.85 (1.35, 4.04)**	**0.0008**	−0.82 (0.49)	0.61 (0.85, 4.02)	0.12
Latino/multi‐racial	−1.06 (0.28)	0.35 (0.70, 2.90)	0.33	−1.89 (0.69)	−0.46 (0.18, 2.23)	0.48
Sex						
Male (Ref)	−0.47 (0.17)	—	—	−0.94 (0.52)	—	—
Female	−1.56 (0.20)	**1.09 (2.12, 4.17)**	**< 0.0001**	−1.81 (0.54)	**0.87 (1.36, 4.19)**	**0.002**
Child opportunity index						
Very low (Ref)	−0.64 (0.32)	—	—	−0.98 (0.79)	—	—
Low	−1.31 (0.43)	0.67 (0.71, 5.40)	0.20	—	—	—
Moderate	−0.34 (0.39)	−0.30 (0.29, 1.92)	0.54	−3.35 (1.71)	2.37 (0.28, 400.53)	0.20
High	−1.41 (0.24)	**0.77 (1.02, 4.60)**	**0.04**	−0.98 (0.47)		0.99
Very high	−1.36 (0.15)	**0.72 (1.09, 3.86)**	**0.03**	−0.21 (0.23)	−0.01 (0.17, 5.89)	0.34
					−0.78 (0.09, 2.24)	
Age	***𝛘*** ^**2**^ **= 66.60**	—	**< 0.0001**	*χ* ^*2*^ = 0.60	—	0.44

^a^ Represents the within‐group differences. Statistically significant differences are bolded.

## 4 Discussion

This study provides evidence that school‐based asthma therapy (SBAT) yields meaningful benefits beyond clinical outcomes [9], particularly in reducing missed instructional time and improving student behavioral functioning. Across a large, diverse sample of students, SBAT enrollment was associated with substantial reductions in total and unexcused absenteeism, alongside improvements in behavioral incidents, although no measurable improvements in grades were observed.

One of the most notable findings is the consistent reduction in missed instructional time following SBAT enrollment. Student absences and missed instructional time are detrimental to learning, with several studies demonstrating negative effects on grades among children with a higher number of unexcused absences [23, 24]. Gershenson et al. reported that unexcused absences were more harmful than excused absences among kindergarten through fifth grade students, and children from low‐income households and English language learners were disproportionately affected. Gottfried [23], using detailed longitudinal data from elementary schools in Philadelphia, investigated how excused and unexcused absences influenced students' performance. Interestingly, greater excused absences demonstrated a positive relationship with achievement, while higher unexcused absences were associated with greater academic risk, particularly in math. An important finding was the marked difference in baseline absenteeism between elementary and middle/high school students. Prior to SBAT enrollment, middle/high school students had substantially higher unexcused missed instructional hours than elementary students, suggesting that older students entered the program with a greater burden of chronic absenteeism. This disparity is consistent with evidence that absenteeism generally increases during adolescence and may reflect greater developmental, behavioral, and social challenges associated with managing chronic diseases such as asthma in older youth. Despite these higher baseline averages, middle/high school students experienced meaningful reductions in unexcused missed instructional hours following SBAT enrollment, indicating that the program was effective even among students with greater initial absenteeism. Although elementary and middle/high school students both benefitted from the program, the higher baseline burden among older students suggests that school‐based asthma therapy may have particular value for adolescents who are at risk for poor attendance.

The observed shift towards fewer unexcused absences, coupled with slight (non‐significant) increases in excused absences, may suggest improved symptom recognition and more appropriate health‐related absenteeism. One possible explanation is that improved asthma management enhanced caregivers' and school personnel's recognition of symptoms warranting an excused school absence. Alternatively, increased awareness of students participating in SBAT may have resulted in more consistent documentation of absences. To examine this alternative explanation, we examined absences coded specifically as medical illness and found that the proportion of all absences attributed to medical illness declined from 1.38% before SBAT enrollment to 0.06% after enrollment, a 96% reduction (*p* < 0.0001). Although this finding cannot definitively distinguish between changes in documentation practices and true changes in health status, the marked reduction in medically coded absences suggests improvements in attendance were accompanied by fewer illness‐related absences. These results align with prior research demonstrating that improved asthma control reduces acute exacerbations and unplanned school absences and extend this work by highlighting SBAT's potential to improve school attendance in real‐world settings.

Importantly, reductions in missed instructional time were consistent across sex and ethnicity, suggesting that SBAT may function as an equitable intervention. Given well‐documented disparities in asthma morbidity and school absenteeism, the ability of SBAT to deliver comparable benefits across demographic groups underscores its potential as a strategy to reduce inequities in both health and academic outcomes.

In addition to attendance, this study found significant improvement in student behavior, particularly for more severe (Level 2) infractions. The overall 32% and 34% reduction in Level 2 incidents for elementary and middle/high school students, respectively, suggest that improved asthma management may translate into better day‐to‐day classroom functioning. Reduced symptom burden, fewer disruptions due to asthma, and improved sleep and overall well‐being may contribute to enhanced self‐regulation and engagement in the classroom. Despite these encouraging findings, no significant improvements were observed in academic grades among middle/high school students. Several factors may explain this result. Academic performance is multifactorial and influenced by a range of factors beyond health and attendance, including prior achievement, instructional quality, and socioeconomic context. It is also possible that the duration of follow‐up (four quarters pre‐ and post‐enrollment) was insufficient to detect measurable changes in grades, particularly if improvements in attendance and behavior require more time to translate into academic gains. Additionally, grades may lack sensitivity to subtle changes in academic engagement or learning, suggesting that future studies should consider alternative academic outcomes such as standardized test scores or measures of classroom participation.

This study has several strengths, including the use of objective, longitudinal school record data and a relatively large sample spanning multiple grade levels. In addition, the Data Use Agreement allowed us to abstract missed instructional minutes, grades, and behavioral incident data in addition to the overall number of excused and unexcused absences.

### 4.1 Implications for School Health, Policy, Practice, and Equity

These findings have important implications for school health, policy, practice, and equity. For school health, SBAT demonstrates that integrating guideline‐based asthma into routine school nursing services can yield meaningful improvements in attendance and student behavior, reinforcing the critical role of school nurses in chronic disease management. From a policy perspective, the observed reductions in school absenteeism and behavioral incidents provide a compelling rationale for investment in and reimbursement for school‐based directly observed therapy programs, particularly as part of broader strategies to improve educational outcomes and reduce health disparities. In practice, schools and districts should consider adopting SBAT as a scalable, pragmatic approach to addressing adherence barriers, with attention to implementation supports such as nurse staffing, care coordination, and partnerships with healthcare providers. Importantly, the consistent benefits observed across sex and ethnicity highlight SBAT's potential as an equity‐promoting intervention, capable of mitigating well‐documented disparities in asthma morbidity, absenteeism, and school engagement among students in disadvantaged, low resource communities.

### 4.2 Limitations

There were several limitations that are noted. The retrospective design and lack of a control group limit causal influence, and unmeasured confounders may have influenced the observed outcomes. The findings reported here, together with those from our previous publication [25] help address this concern by demonstrating that asthma‐related healthcare utilization (likely due to poor symptom control) increased over the 2 years before children enrolled in SBAT leading to the conclusion that there was no improvement demonstrated as a result of natural maturation. Only academic records for children enrolled in one large, urban school district were abstracted and thus, these results may not be generalizable to smaller or rural districts. The absence codes did not allow for specification of asthma‐related absences; thus, we opted to examine excused and unexcused absences. The coding of behavioral incidents by teachers, staff, and administrators can be subjective as it relates to the type of infraction and severity. Furthermore, the absence of Level 3 incidents limited the ability to assess SBAT's impact on more severe disciplinary outcomes. Other factors known to impact school‐age children with asthma such as birthweight, family history of asthma, and household smoking were not available in the academic record database. Because this was not a research study but an implementation evaluation study, we did not conduct surveys with caregivers to obtain this information.

## 5 Conclusions

In summary, this study makes three important contributions to the literature. First, it provides one of the first comprehensive evaluations of academic outcomes associated with SBAT, including excused and unexcused absenteeism, behavioral incidents, and grades. Second, it demonstrates that these outcomes can be achieved through routine implementation rather than under the controlled conditions of a clinical trial. Third, the findings support the integration of guideline‐based asthma management into existing school health workflows as a scalable strategy with the potential to improve both health and educational outcomes.

## Funding

This work was supported by the National Heart, Lung, and Blood Institute (1 R01 HL144652‐01A1).

## Ethics Statement

The study adhered to the Declaration of Helsinki and was approved by the Nationwide Children's Hospital Institutional Review Board (IRB #00000236 Approved 042/2019). Written informed consent/assent was obtained from all caregivers and children. The consent documents were HIPAA and FERPA compliant.

## Conflicts of Interest

Kimberly Arcoleo: NIH research grants; compensation for serving as an external grant reviewer. Holly McGregor: NIH research grants. Phoebe Marx: None. Ian Dela Cruz: None. Elizabeth Allen: None. MaryKay Irwin: Research and program funding received from the Centers for Medicare & Medicaid Services, Ohio Department of Health, and the Governor's Office of Appalachia (part of the Ohio Department of Development). Jill Halterman: NIH research grants; payment for serving on a DSMB. This project was funded by the National Heart, Lung, and Blood Institute (1 R01 HL144652‐01A1) where Dr. Arcoleo served as PI and Drs. McGregor, Allen, Irwin, and Halterman were Co‐Investigators on the grant. None of the other NIH grants listed supported the work reported in this manuscript.

## Data Availability

A de‐identified public use dataset will be deposited with the Inter‐university Consortium for Political and Social Research (ICPSR). In addition, Dr. Arcoleo will make available project‐specific, de‐identified datasets to investigators or graduate students for secondary analysis through completion of a Data Use Agreement.
